# Lightweight Anonymous Authentication for IoT: A Taxonomy and Survey of Security Frameworks

**DOI:** 10.3390/s25175594

**Published:** 2025-09-08

**Authors:** Jian Zhong, Sheng He, Zhicai Liu, Ling Xiong

**Affiliations:** School of Computer and Software Engineering, Xihua University, Chengdu 610039, China

**Keywords:** authentication, Internet of Things, user anonymity, forward secrecy, desynchronization attack

## Abstract

The resource-constrained nature of Internet of Things (IoT) devices necessitates authentication mechanisms built upon lightweight cryptographic primitives, such as symmetric key algorithms and hash functions. In response to demands for user anonymity and forward secrecy, numerous innovative authentication schemes have emerged. This work presents a systematic review of these state-of-the-art approaches. We introduce a structured classification by synthesizing the field into nine distinct sub-frameworks, each focused on either user anonymity or forward secrecy. These are then integrated into two general frameworks that provide both properties. Our analysis illuminates the design principles, security guarantees, and performance trade-offs inherent to each framework. Building on this classification, we comparatively evaluate the security features and performance metrics of 45 representative schemes. Ultimately, this work seeks to enhance the understanding of current challenges and foster further advancement in IoT security.

## 1. Introduction

**Figure 1:**
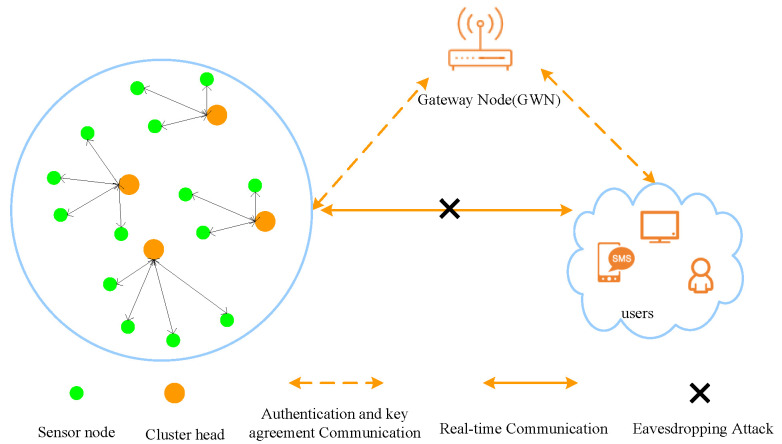
The architecture of the internet of things.

The Internet of Things (IoT) has become a major focus of academic and industrial research over the last two decades, driven by its vast applicability in areas like smart homes, healthcare, and military monitoring. Central to many IoT deployments are Wireless Sensor Networks (WSNs), which, as illustrated in Figure 1, are composed of a gateway node (GWN), external users, and a large-scale deployment of sensor nodes that provide real-time information [1,2]. These nodes are typically distributed, either uniformly or randomly, across a target environment. However, due to the open and wireless nature of communication channels, WSNs are inherently vulnerable to eavesdropping attacks, whereby adversaries intercept transmitted data to obtain sensitive information without detection.

The extensive and intricate real-time data generated by IoT introduce significant security and privacy challenges, particularly in authenticating external users to IoT-enabled sensor nodes [3]. Mutual authentication is therefore indispensable for ensuring that only authorized users can access trustworthy nodes. However, conventional authentication protocols are vulnerable to identity masquerading and tracing attacks, making user anonymity, encompassing both identity protection and untraceability, a vital security requirement [4,5]. Furthermore, sensor nodes are often deployed in unattended, hostile environments and typically lack tamper-resistant hardware. This leaves their stored long-term private keys susceptible to physical compromise by adversaries. Such a compromise threatens the confidentiality of all previous session keys, thereby jeopardizing the long-term security of the IoT ecosystem [6]. Consequently, forward secrecy, the property that prevents the exposure of long-term keys from compromising past session keys, has emerged as another critical security attribute for IoT frameworks.

Developing an anonymous authentication scheme with forward secrecy for the IoT environment is a critical yet challenging endeavor. While research has shown that public-key cryptography is often necessary to achieve genuine user anonymity and forward secrecy [7,8,9], the resource constraints of most sensor nodes present a significant hurdle to its adoption. Consequently, there is a strong preference for lightweight primitives like symmetric ciphers and hash functions in IoT authentication protocols [10]. This creates a central design tension: how to construct a robust authentication scheme that guarantees both user anonymity and forward secrecy using only these lightweight building blocks. Addressing this formidable challenge is the primary focus of the works reviewed in this paper.

To meet these stringent security requirements, a multitude of lightweight anonymous authentication protocols have been proposed, leveraging solely symmetric ciphers and hash functions. These protocols have made significant strides in addressing security and privacy challenges across various IoT environments. For instance, techniques like dynamic pseudonym IDs and one-time hash chains have become cornerstones for achieving user anonymity and forward secrecy, respectively [11,12]. While effective, these dynamic techniques introduce potential challenges, most notably the risk of desynchronization if messages are lost or blocked by an attacker. However, robust protocol design typically mitigates this issue through built-in recovery mechanisms. This survey delves into these strategies, analyzing how different frameworks ensure both security and resilience against such operational disruptions.

This study presents an in-depth analysis of lightweight anonymous authentication schemes for the IoT developed over the past decade. Our primary contribution is the distillation and synthesis of generic authentication frameworks that underpin these schemes. A key focus is on the strategies devised to mitigate desynchronization attacks, a common vulnerability in dynamic protocols. Through systematic analysis and comparison, we elucidate the advantages and disadvantages of each framework, thereby providing a theoretical foundation to guide the design of future protocols. Specifically, we scrutinize various asynchronous mitigation techniques to offer insights into optimizing the security, efficiency, and reliability of authentication in IoT.

### 1.1. Our Contributions

This study makes the following specific contributions to the field of lightweight anonymous authentication for the IoT:

- We propose a novel taxonomy by deconstructing existing schemes into nine fundamental sub-frameworks. We analyze six sub-frameworks designed for user anonymity and three for forward secrecy, elucidating their core mechanisms and design principles.
- We synthesize these components into two general frameworks that concurrently provide both user anonymity and forward secrecy. These overarching models offer robust, reusable blueprints for developing new, secure authentication protocols.
- We conduct a comprehensive comparative analysis of all proposed frameworks. This includes an evaluation of their strengths and limitations, as well as their resilience to key security threats such as desynchronization attacks while also considering performance and multi-factor security capabilities.

### 1.2. Organization of the Paper

The remainder of this paper is organized as follows. Section 2 reviews related work in lightweight IoT authentication. Section 3 establishes the system model and security definitions used throughout our analysis. In Section 4, we deconstruct existing protocols into nine distinct sub-frameworks, detailing their architecture and functionality. Section 5 synthesizes these into two general frameworks for anonymous and forward-secure authentication. Section 6 presents a comparative security and performance analysis of these frameworks. Finally, Section 7 concludes the paper with a summary of our findings and their implications.

## 2. Overview of Related Work

**Figure 2:**
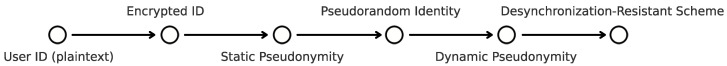
Evolution of lightweight anonymous authentication schemes for IoT.

Over the past decade, a multitude of lightweight anonymous authentication schemes have been proposed for the IoT. This section reviews the evolution of these schemes, focusing primarily on the pivotal security goals of user anonymity and forward secrecy. To illustrate this evolution, Figure 2 presents a chronological progression of representative techniques, from plaintext identifiers to desynchronization-resistant schemes. We summarize the key technical advancements that have shaped the design of modern authentication mechanisms for IoT ecosystems.

Early lightweight authentication schemes for the IoT, such as the one proposed by Wong et al. in 2006 [13], often overlooked user anonymity while also suffering from vulnerabilities like replay and stolen-verifier attacks [14]. Several subsequent schemes, including [14,15,16,17,18], attempted to provide anonymity by using a shared secret parameter to mask user identities. However, because this secret was common to all users, these schemes failed to achieve true anonymity, as any legitimate user could impersonate another. To provide genuine user anonymity, a secret must be shared exclusively between the sender and receiver. Many modern schemes [19,20,21,22,23,24] adopt this principle by encrypting the user’s real identity. While this approach protects against identity disclosure, it introduces a significant practical challenge for the receiver (e.g., a gateway node). Without knowing the sender’s identity beforehand, the receiver must perform an exhaustive search, attempting to decrypt the message with every possible user’s key until the correct one is found. This computational overhead makes such a “search-by-decryption” approach impractical for large-scale IoT systems [11].

To overcome the impracticality of exhaustive search operations, many schemes [25,26,27,28,29] employ a static pseudonym ID during authentication. This pseudonym can either serve as a direct pointer to the user’s real identity in a database or be decryptable to reveal the real ID [30,31,32,33,34,35,36,37]. While this approach prevents direct identity disclosure, the consistent use of a static ID across sessions makes users traceable, thus failing to provide untraceability, a critical aspect of user privacy. To address this limitation, subsequent research introduced dynamic pseudonym IDs [38,39,40,41,42,43,44,45], which are updated after each successful authentication session. By ensuring that pseudonyms vary over time, these schemes effectively thwart user tracking. However, this dynamism introduces a new vulnerability: desynchronization attacks. If an adversary intercepts and blocks an ID update message, the sender’s and receiver’s states become desynchronized, potentially rendering the protocol inoperable until a manual re-registration process is performed [46]. This highlights the critical need for robust synchronization-recovery mechanisms in dynamic authentication systems.

At present, three primary approaches exist to mitigate desynchronization attacks in dynamic ID schemes. The first involves unilateral ID updates, where schemes like [47,48,49] use a derived key for authentication to avoid desynchronization. This method provides session-level anonymity by allowing client devices to dynamically update pseudonyms. Recipients can reconstruct a user’s real identity using these pseudonyms should network disruptions interrupt message delivery, thus solving asynchronous communication identity authentication challenges. Its core advantages are lightweight implementation, requiring only pseudonym update logic, and operational efficiency that balances anonymity with low computational overhead. However, a critical vulnerability arises: transmission interruptions may prevent timely pseudonym updates, causing subsequent messages to reuse outdated identifiers. This creates logical links between disjointed communications. A second, more robust approach, proposed by Gope et al. [11,50,51,52,53], equips users with a pre-allocated set of emergency IDs for resynchronization. However, this technique incurs significant storage overhead and requires a manual re-registration process once the emergency credentials are exhausted. Third, a more resource-efficient approach, like schemes in [54,55,56], requires the receiver to store only two pseudonyms: the current one and the previous one. This small state window allows the system to recover from a single lost update message, effectively balancing resilience against desynchronization with minimal storage requirements.

While preceding analysis focused on user anonymity, achieving forward secrecy in lightweight schemes introduces similar challenges, particularly regarding desynchronization. For instance, in protocols that use one-time hash chains for forward secrecy, such as Gope and Hwang’s scheme [11], a blocked message can cause the hash values on the sender and receiver sides to become desynchronized. Therefore, a principal challenge in IoT security is designing a lightweight mechanism that concurrently provides user anonymity, forward secrecy, and resilience to desynchronization attacks. Several recent schemes have attempted to meet these combined requirements. However, many fall short: for example, Shuai et al.’s scheme [40] focuses only on the desynchronization of forward secrecy, while Yang et al.’s scheme [25] fails to provide user anonymity. Although some protocols fulfill all three security goals [55,57,58], they often do so at the cost of high communication overhead, requiring five message rounds. Seeking to improve this, Xiong et al. [56] proposed a secure four-round scheme, demonstrating a clear trade-off between security completeness and protocol efficiency.

## 3. System Model and Definitions

This section provides an overview of the system architecture, delineates the adversary model, and outlines the security requirements pertaining to authentication mechanisms within the realm of IoT.

### 3.1. System Model

**Figure 3:**
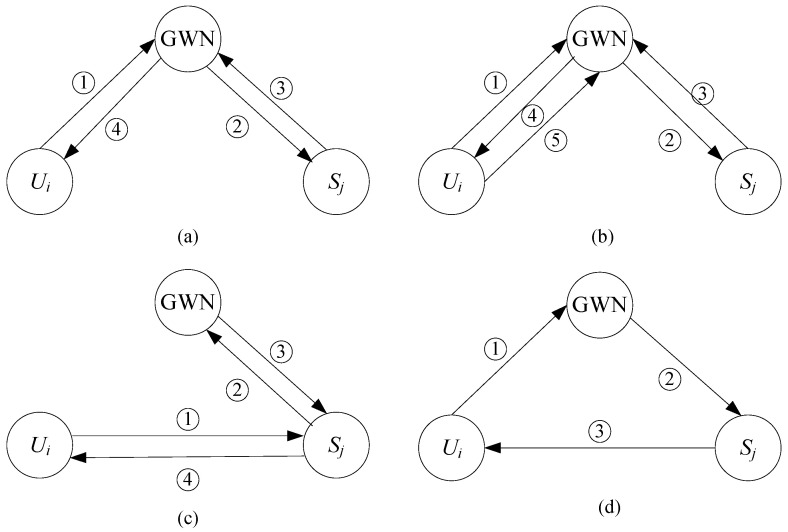
Four basic system architectures for authentication in IoT environments.

In IoT ecosystems, the authentication framework typically involves three primary entities: the external user $U_{i}$, the sensor node $S_{j}$, and the gateway node (GWN). The GWN functions as a trusted third party, tasked with generating secure parameters. The sensor node $S_{j}$ processes data for authenticated users, while $U_{i}$ accesses real-time data from the target sensor node $S_{j}$. As illustrated in Figure 3, four prevalent architectures for IoT authentication schemes are summarized. Model (a), adopted in [56,59], is the most widely used. Model (b), an extension of Model (a) incorporating an additional message exchange between $U_{i}$ and GWN, is represented in [55]. Models (c) and (d), described in [37] and [34,60], respectively, are common in earlier schemes but are susceptible to vulnerabilities such as GWN bypassing and Denial of Service (DoS) attacks, as noted in [61]. Models (a) and (b) are the primary research focus due to their performance-security trade-off. They partition the IoT system into three communication paths: $U_{i}$ to GWN, GWN to $S_{j}$, and $U_{i}$ to $S_{j}$. This work addresses the challenge of ensuring user anonymity, forward secrecy, and asynchronous attack resistance across these pathways.

### 3.2. Adversary Model

Building on prior work [55,56], we introduce a refined adversary model. In this model, the adversary *A* is assumed to have the following capabilities:

- *A* can intercept, modify, and replay messages transmitted between users, sensor nodes, and the GWN.
- *A* can compromise a user’s smart device to extract confidential parameters from their smart card, such as the password validation value. This enables *A* to perform an offline password guessing attack.
- *A* can compromise a sensor node and extract its stored confidential parameters.
- *A* can obtain previously established session keys.
- *A* can masquerade as a legitimate user.
- *A* can impersonate a legitimate sensor node.

### 3.3. Adversary Model

Given that communication channels are characterized by openness and wirelessness, wireless sensor networks are inherently vulnerable to eavesdropping attacks. The adversary A has two goals. One is forging authentication message among $U_{i}$, $S_{j}$, and GWN. The other is to obtain the session key between $U_{i}$ and $S_{j}$. We assume *A* is a adversary with probabilistic polynomial time. The feasible attack capabilities of *A* are summarized as follows:

- *A* can fully control the two communication channels amongthe user, the sensor and the GWN, which means that A isable to intercept, modify, or block messages transmitted in the public channel.
- *A* is capable of compromising another legitimate external user in the system.
- *A* is capable of compromising another legitimate sensornode in the system.

### 3.4. Security Requirements

Numerous security properties for lightweight authentication protocols in the IoT have been discussed in the literature [55,56,61,62]. This work focuses on achieving the following key security guarantees and features: mutual authentication, user anonymity (providing both untraceability and unlinkability [4,5]), forward secrecy, multi-factor security, efficient GWN discovery, and resistance to desynchronization attacks.

- **Mutual Authentication:** A protocol provides mutual authentication if it ensures that a probabilistic polynomial-time (PPT) adversary cannot successfully impersonate a legitimate user, sensor node, or the GWN.
- **User Anonymity:** This property comprises two components: untraceability and unlinkability [4,5].
  - **Untraceability** ensures that a PPT adversary cannot determine a user’s real identity from intercepted messages.
  - **Unlinkability** ensures that a PPT adversary cannot link multiple sessions or messages to the same user. A secure protocol must protect against both, as even protocols with dynamic pseudonyms can be vulnerable if other static identifiers are transmitted.
- **Forward Secrecy:** This property ensures that the compromise of long-term secrets (e.g., a user’s password or a sensor’s private key) does not compromise the confidentiality of past session keys. Even if an adversary *A* obtains these long-term secrets, they cannot use them to decrypt previously captured communication.
- **Multi-factor Security:** A scheme is considered multi-factor secure if it remains secure even when an adversary compromises some, but not all, of its authentication factors (e.g., n−1 out of *n* factors) [6,63,64]. For a typical three-factor scheme (password, smart card, and biometrics), this means an adversary still cannot impersonate a user after compromising any two of the three factors [8,65].
- **Efficient Identity Lookup:** Since users’ real identities are concealed to ensure anonymity, the GWN must have an efficient mechanism to look up a user’s or sensor’s credentials from the temporary identifiers they present during authentication. This lookup operation must not become a performance bottleneck for the system.
- **Resistance to Desynchronization Attacks:** A desynchronization attack occurs when an adversary blocks messages, causing a mismatch in state variables (e.g., counters or one-time nonces) between communicating parties. A resistant protocol must be self-synchronizing, ensuring that such a temporary mismatch does not prevent the parties from successfully authenticating in a subsequent session.

### 3.5. System Building

The discussed schemes primarily utilize two cryptographic primitives: a symmetric encryption algorithm (*E*) and a Message Authentication Code (MAC). These are specified as follows:

**Symmetric Encryption:** In symmetric encryption, a single secret key is shared between communicating parties for both encryption and decryption. We denote the encryption of a message *M* with key *k* as $E_{k}M$. In the protocols we review, this encryption is typically implemented in one of two ways:

- **Symmetric Cipher-Based Encryption:** This is the most common approach, using standard block ciphers like AES, 3DES, or DES to implement $E_{k}M$. As these algorithms are well documented in the literature, their technical specifications are not detailed here.
- **Hash Function-Based Encryption:** This method achieves encryption by XORing the message *M* with a key stream derived from a cryptographic hash function (e.g., SHA-256). For instance, in the scheme by Yang et al. [25], the sensor’s identity $sid_{j}$ is encrypted as
  (1) $$E_{ac_{id_{i}}}\left\{sid_{j}\right\}=h(ac_{id_{i}}\left|\right|N_{id_{i}}\left||r\right)⊕sid_{j}$$
  Here, the key stream is generated by hashing the shared secret $ac_{id_{i}}$ with a fresh nonce $N_{id_{i}}$ and a constant *r*. This approach is well suited for IoT environments where plaintexts are often short (e.g., identities or nonces). Furthermore, since a hash operation is significantly faster than a symmetric cipher operation (e.g., at least 10 times faster in software [66]), protocols using this hash-based approach are generally more lightweight.

**Message Authentication Code (MAC):** A MAC is a cryptographic primitive that provides both message integrity and authenticity. It is a short piece of information used to verify that a message came from the stated sender (authenticity) and has not been altered in transit (integrity).

In lightweight protocols, a MAC is commonly implemented as a Hash-based MAC (HMAC). The process is as follows: the sender uses a shared secret key to compute a MAC value, often called a tag, for the message. This tag is then appended to the message before transmission.

Upon receiving the message and its tag, the receiver re-computes the tag on the received message using the same shared key. If the re-computed tag matches the received tag, the receiver can be confident that:

- The message has not been tampered with, as any modification would result in a mismatched tag;
- The message originated from a party possessing the secret key, since an adversary without the key cannot forge a valid tag.

Thus, a MAC is a fundamental mechanism for defending against message modification and sender impersonation.

## 4. The Taxonomy of Nine Sub-Frameworks

In this section, we introduce a framework consisting of nine sub-frames designed to provide either user anonymity (UA) or forward secrecy (FS). The sub-frames are labeled as follows: SF1-UA through SF6-UA for user anonymity, and SF7-FS through SF9-FS for forward secrecy. These sub-frames are applied across the three communication paths defined in Models (a) and (b), shown in Figure 3. Table 1 summarizes the notations used.

### 4.1. SF1-UA

#### 4.1.1. Review SF1-UA

SF1-UA is widely used in IoT authentication schemes. It ensures user anonymity by embedding the sender’s actual identity within ciphertext. Based on the origin of the secret parameters involved, SF1-UA can be classified into two distinct categories.

- The secret key used for protecting the real identity is a shared key of the sender and the receiver (see [19,21,22,23,24]). Takeing Wazid et al.’s scheme [21] as an example, the shared secret key is known only to the user and GWN. Using this key stored in its database, the GWN can retrieve the user’s real identity.
- The secret key used for protecting the real identity is shared by all numbers in the system (see [14,15,16,17,18]. For example, in Tai et al.’s scheme [15], each user’s smart card stores the shared secret key with the GWN. When the GWN receives the ciphertext of identity, it can decrypt and get the real Identity.

**Figure 4:**
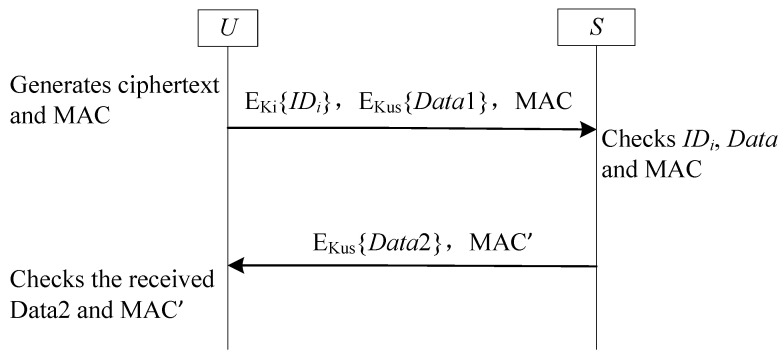
The sub-framework SF1-UA for user anonymity.

Figure 4 depicts the SF1-UA process framework used in communication paths of Models (a) and (b) from Figure 3: $U_{i}$ to GWN, GWN to $S_{j}$.

#### 4.1.2. Analysis SF1-UA

The first category of SF1-UA protocols effectively preserves identity confidentiality by resisting disclosure attacks. This approach encrypts the user’s identity together with a timestamp during each authentication session, preventing identity linking through cryptographic freshness. Within IoT environments where sensor nodes store a unique GWN secret key, this SF1-UA variant remains suitable for GWN-to-sensor-node communications (Model (b) in Figure 3). However, for channels between $U_{i}$ and GWN (Models (a) and (b)), this method necessitates impractical search operations at the GWN. As each user’s secret key is stored in the GWN’s information table, the gateway cannot directly associate incoming identity ciphertexts with specific users. Following the design by Wazid et al. [21], the GWN must consequently perform exhaustive searches across all user keys to identify the correct communication partner after receiving such ciphertexts. Therefore, this SF1-UA category proves optimal for anonymous protection exclusively in GWN-to-$S_{j}$ paths; Figure 3.

As for the second category of SF1-UA, the GWN can distinguish the exact user by its secret key; the problem of the impractical GWN search operation is not occurring. However, since each user in the system has this key and the real identity can be recovered by the member in the system, this method can protect identity from external attackers, not internal members. Like the above Tai et al.’s scheme [15], every internal member can obtain the real identify via decrypting the ciphertext of identity. Therefore, this kind of SF1-UA is only suitable for a trusted system where every member is not malicious.

### 4.2. SF2-UA for User Anonymity

#### 4.2.1. Review SF2-UA

SF2-UA addresses the search inefficiency in $U_{i}$-to-GWN communications that affects SF1-UA protocols. This approach incorporates static pseudonymous identifiers during message transmission, eliminating exhaustive key searches at the GWN. Based on implementation mechanisms, SF2-UA implementations fall into two distinct categories.

- Static pseudonym identifiers stored in the receiver’s information table provide a mapping to the sender’s real identity (see [25,26,27,28,29]). For instance, in Yang et al.’s scheme [25], a shared static pseudonym serves as the reference identifier between $U_{i}$ and the GWN. The gateway uses this pseudonym to retrieve the corresponding user’s real identity and secret key.
- Retrieving the real identity requires decrypting transmitted data using both the static pseudonym identifier and the receiver’s long-term secret key (see [30,31,32,34,35,36,37]). Ostadsharif et al.’s scheme [30] exemplifies this approach: upon receiving a user message, the gateway decrypts the embedded identity using its stored pseudonym identifier.

**Figure 5:**
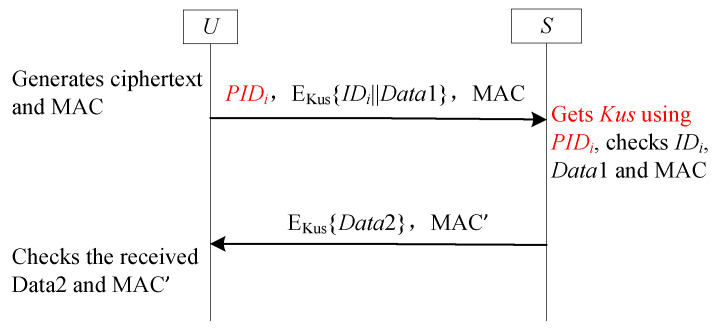
The sub-framework SF2-UA for user anonymity.

Figure 5 depicts the SF2-UA process framework used in communication paths of Models (a) and (b) from Figure 3: $U_{i}$ to GWN.

#### 4.2.2. Analysis SF2-UA

SF2-UA resolves GWN search inefficiency by enabling receivers to retrieve real identities directly from static pseudonyms. However, both SF2-UA variants remain vulnerable to identity tracking: adversaries can correlate user activities using the fixed pseudonym identifiers. Consequently, SF2-UA is only viable in environments where user linkability attacks are inconsequential.

### 4.3. SF3-UA for User Anonymity

#### 4.3.1. Review SF3-UA

**Figure 6:**
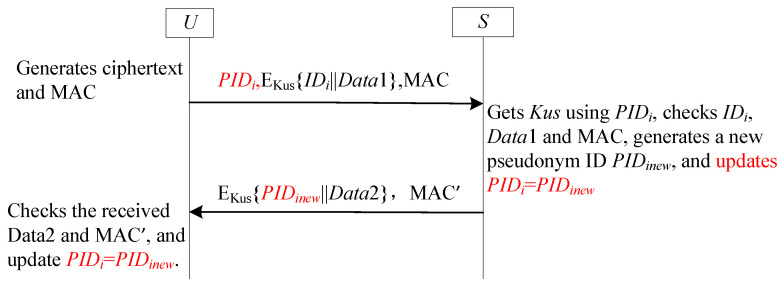
The sub-framework SF3-UA for user anonymity.

SF3-UA addresses identity tracking vulnerabilities by employing dynamically updated pseudonyms. Both communicating entities refresh their pseudonym identifiers after successful sessions (see [38,39,40,41,42,43,44,45]). As implemented in Wazid et al.’s scheme [39], the gateway uses these session-bound pseudonyms to retrieve the user’s real identity and secret key from its database. Figure 6 details this operational workflow.

#### 4.3.2. Analysis SF3-UA

SF3-UA prevents both identity disclosure and tracking through its dynamic pseudonyms. However, if an adversary compromises the returned message in Figure 6, synchronization failures may propagate across both endpoints. Consequently, SF3-UA deployment should be restricted to synchronization-sensitive IoT environments.

### 4.4. SF4-UA for User Anonymity

#### 4.4.1. Review SF4-UA

Unlike SF3-UA, SF4-UA eliminates synchronization vulnerabilities by restricting pseudonym updates to unilateral modifications (user-exclusive), with real identities encrypted using a derived key synthesized from the current pseudonym identifier and receiver’s long-term secret key [47,48,49,67,68]. As implemented in Banerjee et al.’s scheme [67], upon receiving $U_{i}$’s message, the GWN recovers the real identity by decrypting the ciphertext using the dynamic pseudonym and its long-term key. Post-authentication, the gateway generates a fresh random number as the new dynamic pseudonym, computes a corresponding derived key, and transmits both components to $U_{i}$, which replaces its outdated identifiers while the GWN maintains existing credentials-enforcing user-side-exclusive updates that inherently prevent desynchronization.

**Figure 7:**
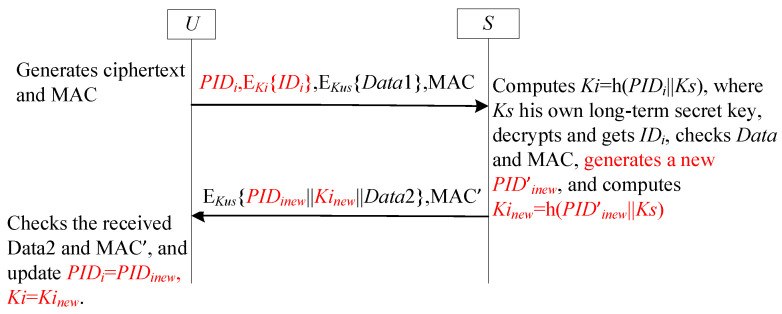
The sub-framework SF4-UA for user anonymity.

Figure 7 details this protocol workflow, where Ki is the derived key generated to protect the real identity $ID_{i}$ (Ki is the hash value of pseudonym ID $PID_{i}$ and *S*’s long-term secret key $K_{s}$), $K_{us}$ is the shared key between *U* and *S*, $K_{s}$ is the long-term secret key of *S*.

#### 4.4.2. Analysis SF4-UA

SF4-UA’s unilateral update mechanism resolves desynchronization vulnerabilities inherent in anonymous authentication systems. By restricting pseudonym updates to one endpoint while enabling verification via the counterpart’s long-term key, SF4-UA maintains consistent pseudonym state synchronization despite network delays or timing variances. This architecture establishes SF4-UA as a robust solution to desynchronization threats in user anonymity frameworks.

### 4.5. SF5-UA for User Anonymity

#### 4.5.1. Review SF5-UA

**Figure 8:**
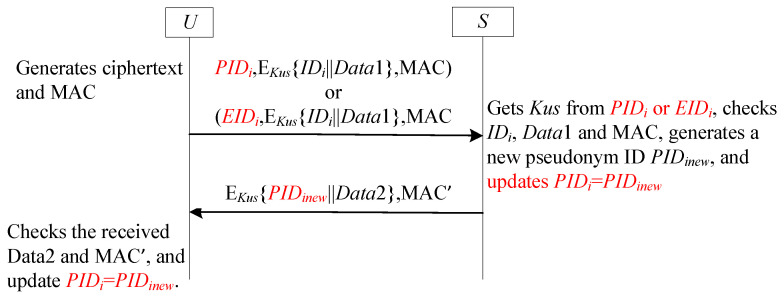
The sub-framework SF5-UA for user anonymity.

To mitigate SF3-UA’s synchronization flaws, SF5-UA provisions pre-installed emergency keys at both communication endpoints. These contingency keys trigger automatic resynchronization following detected failures [11,50,51,52,53], with Figure 8 formalizing the protocol sequence. In Figure 8, $EID_{i}$ denotes the pre-established emergency pseudonym identity stored at both U and S endpoints, deployed during desynchronization attacks.

#### 4.5.2. Analysis SF5-UA

Integrating dynamic pseudonyms with emergency key sets optimizes desynchronization attack resistance in lightweight anonymous authentication. This dual mechanism provides enhanced privacy protection through evolving pseudonyms preventing long-term tracking and guaranteed continuity via emergency fail-safes maintaining synchronization despite failures. Together, these complementary components form a defense-in-depth architecture resilient against synchronization threats.

### 4.6. SF6-UA for User Anonymity

#### 4.6.1. Review SF6-UA

**Figure 9:**
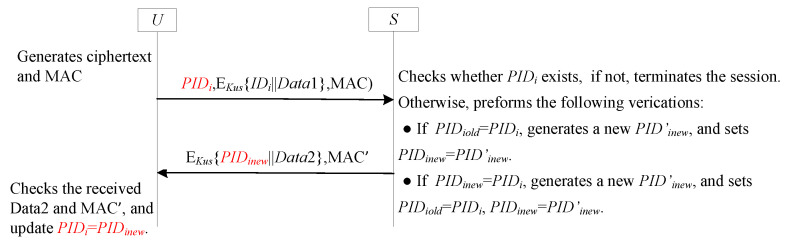
The sub-framework SF6-UA for user anonymity.

The SF6-UA framework provides a resource-efficient countermeasure against desynchronization attacks, eliminating large emergency key requirements. As exemplified by Chang et al. [54], the gateway preserves dual pseudonym values per user: $PID_{inew}$ for the expected new pseudonym and $PID_{iold}$ for the previously used one. This dual-value maintenance enables user recognition when failed updates cause transmission of $PID_{iold}$ instead of $PID_{inew}$. Figure 9 formalizes this protocol’s operational mechanics.

#### 4.6.2. Analysis SF6-UA

The SF6-UA framework preserves user anonymity through session-specific dynamic pseudonyms while also ensuring strong resistance to desynchronization attacks, as illustrated in Figure 9. This resilience manifests in two attack scenarios. First, if an adversary blocks the initial message from the user, no state change occurs on either side, thus preventing any desynchronization. Second, if the adversary blocks the return message from the receiver (e.g., the GWN), the user’s pseudonym ID is not updated while the receiver’s state advances. This creates a temporary desynchronization. Nevertheless, the protocol remains operational due to the receiver’s retention of the previous pseudonym, $PID_{iold}$. Upon receiving the same pseudonym in the subsequent session, the receiver authenticates it against $PID_{iold}$, thereby restoring synchronization. This mechanism enables provable recovery from desynchronization attacks.

### 4.7. SF7-FS for Forward Secrecy

#### 4.7.1. Review SF7-FS

**Figure 10:**
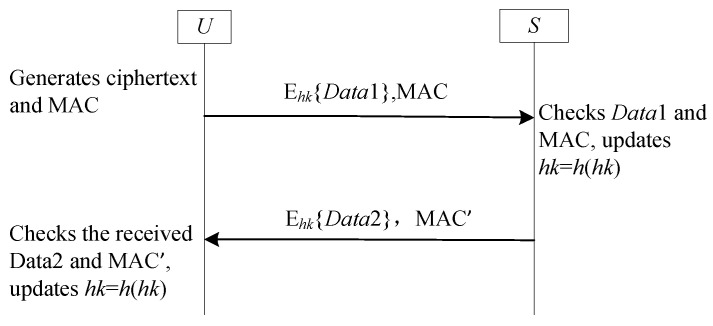
The sub-framework SF7-FS for forward secrecy.

The SF7-FS framework implements forward secrecy in lightweight authentication through evolving session secrets, adapting the one-time hash value concept from Nali et al. [69]. $U_{i}$ and GWN share a secret seed value that undergoes incremental hashing after each successful session (e.g., [11,38]), generating chained one-time hash values to protect past communications. Figure 10 formalizes this operational workflow, where hk denotes a sessionally updating secret key shared between *U* and *S*.

#### 4.7.2. Analysis SF7-FS

As illustrated in Figure 10, the SF7-FS framework provides forward secrecy. Even if an adversary compromises a participant and obtains the current one-time hash value hk, they cannot derive any previous session keys. This is because hk is updated after each successful session via a one-way function, making past values computationally infeasible to recover. However, similar to the SF3-UA framework, SF7-FS is vulnerable to desynchronization attacks. If an adversary blocks the returned message, *S* will have updated its hash value while *U* has not. This desynchronization of the shared secret hk causes subsequent authentication attempts to fail.

### 4.8. SF8-FS for Forward Secrecy

#### 4.8.1. Review SF8-FS

**Figure 11:**
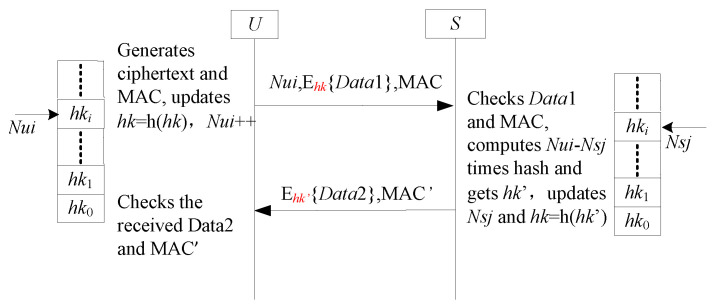
The sub-framework SF8-FS for forward secrecy.

The SF8-FS framework addresses the desynchronization vulnerability of SF7-FS by introducing a serial number to track updates to the one-time hash value hk. As illustrated in Figure 11, each participant maintains a counter (e.g., $N_{ui}$ for *U*, $N_{sj}$ for *S*), initialized to zero. This serial number is incremented with each update to the hash chain, effectively recording the number of hash operations performed (e.g., [40,55,56,57,58]). A critical aspect of this design is that *U* must update its hash value and increment its serial number before transmitting a message, ensuring *S* can always calculate the state difference and resynchronize if necessary.

#### 4.8.2. Analysis SF8-FS

The SF8-FS framework mandates that *U* updates its hash value hk and its serial number $N_{ui}$ before sending message. This design ensures resilience against two primary desynchronization scenarios. First, we consider an adversary that blocks the initial message from *U* to *S*. In this case, *U*’s state (hk, $N_{ui}$) has advanced, while *S*’s state remains unchanged. When *S* receives this message in a subsequent attempt, it detects a discrepancy between its own serial number, $N_{sj}$, and the received $N_{ui}$. Using the difference, $\Delta N=N_{sj}-N_{ui}$, *S* can compute the correct, synchronized hash value by applying the hash function ΔN times to its own hk. The unidirectionality of the hash function prevents *S* from reverting to past states. Second, if an adversary blocks the response message from *S* to *U*, both parties will have already successfully updated their respective states. The protocol has effectively completed for that session, and the loss of the final message does not impact the synchronization of future sessions. Therefore, the SF8-FS framework provides a robust method for preventing desynchronization attack while maintaining forward secrecy.

### 4.9. SF9-FS for Forward Secrecy

#### 4.9.1. Review SF9-FS

**Figure 12:**
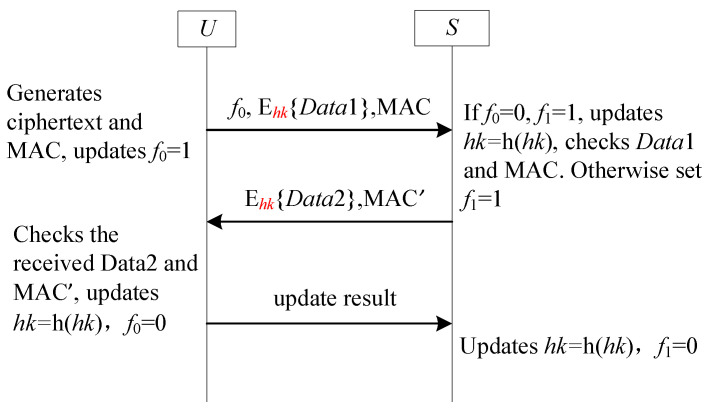
The sub-framework SF9-FS for forward secrecy.

This section summarizes another sub-frame based on the tag technique to achieve forward secrecy. Unlike those based on the serial number technique, the sender *U* and the receiver *S* store tags $f_{0}$ and $f_{1}$ with an initial value of 1, which will be changed to 0 after the one-time hash chain value is updated. Yang et al.’s scheme [25] falls into this category. Figure 12 illustrates this sub-frame.

#### 4.9.2. Analysis SF9-FS

SF9-FS is designed to address the problem of desynchronization attack. Here, we outline how to resist to desynchronization attack. All malicious scenarios launched by the adversary summarize the following possible combinations of $f_{0}$ and $f_{1}$.

- **Combination 1:**$f_{0}=1$ and $f_{1}=1$. In this scenario, the hash chain values of both sides are not updated. So, this combination is omitted.
- **Combination 2:** $f_{0}=0$ and $f_{1}=0$. In this case, the hash chain values of $U_{i}$ and GWN are updated. So, this combination is omitted.
- **Combination 3:** $f_{0}=1$ and $f_{1}=0$. This scenario is impossible because the hash chain values of *A* update first. If $f_{0}=1$, the value of $f_{1}$ must be 1. Therefore, this combination is omitted.
- **Combination 4:** $f_{0}=0$ and $f_{1}=1$. In this scenario, *U* updates its hash chain value while *S* does not. Thus, the hash chain values of *U* and *S* do match each other. This scenario does not cause SF9-FS to be completely unusable because we use $f_{0}$ and $f_{1}$ to record whether the hash chain value has been updated. When *S* finds the asynchronous by checking the value of $f_{1}$, it can update the hash chain value. Accordingly, this scenario may cause asynchronous between *U* and *S*, but it does not have any impact on the future session.

## 5. Two General Frameworks

The protocol architecture comprises six user anonymity sub-frameworks (SF1-UA to SF6-UA) and three forward secrecy sub-frames (SF7-FS to SF9-FS). Initially, each sub-frame implements a single dedicated security function. Theoretically, these anonymity and forward secrecy sub-frames can be combined in 6×3=18 distinct configurations to construct authentication schemes satisfying both requirements concurrently (based on the three communication paths defined in Models (a) and (b) of Figure 3). However, directly combining these functionally distinct sub-frames is non-trivial. Naive integration may result in increased computational overhead, security vulnerabilities from protocol incompatibilities, or suboptimal resource utilization. Consequently, appropriate adjustments and optimizations are essential to ensure the resulting composite frameworks are both secure and operationally efficient. To demonstrate this process, SF6-UA (representative user anonymity sub-frame) and SF9-FS (representative forward secrecy sub-frame) were selected. Two general frameworks, GF1 and GF2, were developed to simultaneously meet both security requirements. GF1 is constructed by integrating SF6-UA and SF9-FS. GF2 is an optimized variant of GF1, specifically enhancing communication performance by replacing the three-round exchange with a single-round protocol.

### 5.1. GF1

#### 5.1.1. Review GF1

**Figure 13:**
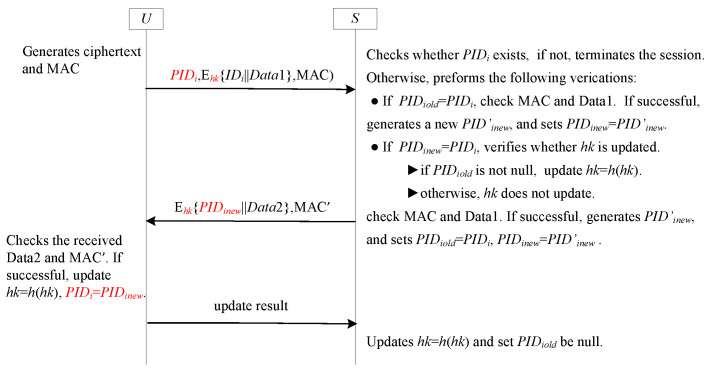
GF1 with three rounds for user anonymity and forward secrecy.

The GF1 framework, leveraging the benefits of both SF6-UA and SF9-FS, provides concurrent support for both user anonymity and forward secrecy. This capability effectively mitigates the issue of desynchronization. However, it is not a direct combination of the SF6-UA and SF9-FS. Instead, it integrates the core SF6-UA components with the essential SF9-FS elements, leveraging their mechanisms. This integration process incorporates specific refinements. Figure 13 depicts the specific steps involved in the implementation of the GF1 framework. In Figure 13, $PID_{i}$ denotes the *U*-stored pseudonym ID. The *S* maintains two pseudonym IDs: $PID_{iold}$ and $PID_{inew}$. Similar to SF6-UA, $PID_{inew}$ stores the new pseudonym ID while $PID_{iold}$ contains the previous one. Within the GF1 framework, however, $PID_{iold}$ has a simultaneous role: it functions both as a random number and as a label for hash-chain updates. The state of $PID_{iold}$ is null; it implies that the one-time hash chain value has already been updated during the previous session. Otherwise, the one-time hash chain value remains unchanged. At initialization, $PID_{inew}$ is set equal to $PID_{i}$, and $PID_{iold}$ is initialized as null.

#### 5.1.2. Analysis GF1

GF1 is an exceptionally well-suited framework for deployment in communication channels that connect user entities, denoted as $U_{i}$ (similar to *U* depicted in Figure 13) and the GWN (analogous to the receiver *S* illustrated in Figure 13), particularly in IoT environments. A number of recent research schemes, such as those presented in [55,57], can be classified within the GF1 framework. For a detailed security analysis of GF1, readers are referred to the relevant literature, including [55,57]. GF1 effectively achieves user anonymity and forward secrecy while mitigating the risk of desynchronization. Nevertheless, in contrast to the majority of two-round lightweight authentication schemes, GF1 requires three rounds to accomplish the authentication process. Although this additional round enhances security functionality, it also incurs extra communication costs.

To quantify this overhead, we assume the bit length of PID is 32 bits, ID is 32 bits, data payload (${\mathrm{Data}}_{1}$, ${\mathrm{Data}}_{2}$) is 128 bits each, and MAC is 256 bits.

**Round 1 (U → S):** The message $\left\{PID_{i},E_{hk}\left(ID_{i}\;∥\;{\mathrm{Data}}_{1}\right),MAC\right\}$ requires (32+160+256)=448 bits, where $E_{hk}\left(ID_{i}\;∥\;{\mathrm{Data}}_{1}\right)$ denotes the encryption of a 32-bit ID and a 128-bit data payload, resulting in 160 bits of ciphertext, and MAC is 256 bits.

**Round 2 (S → U):** The message $\left\{E_{hk}\left(PID_{inew}\;∥\;{\mathrm{Data}}_{2}\right),MAC^{\prime }\right\}$ requires (160+256)=416 bits, where $E_{hk}\left(PID_{inew}\;∥\;{\mathrm{Data}}_{2}\right)$ consists of a 32-bit $PID_{inew}$ and a 128-bit ${Data}_{2}$.

**Round 3 (U → S):** The message {updateresult} requires 32 bits, assuming a minimal acknowledgment payload.

Therefore, the total communication cost per authentication session is 896 bits.

### 5.2. GF2

#### 5.2.1. Review GF2

**Figure 14:**
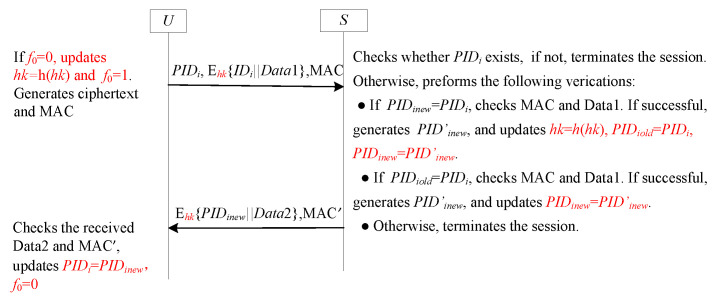
GF2 with two rounds for user anonymity and forward secrecy.

To streamline the authentication process and minimize the number of communication rounds, a novel general framework called GF2 has been introduced [56]. This framework integrates a tag mechanism along with two dynamic pseudonym ID techniques. It is specifically designed for deployment in the communication channel between the user (akin to the sender *U* shown in Figure 14) and the GWN (similar to the receiver *S* in Figure 14).

A detailed illustration of the GF2 framework is provided in Figure 14, which visually demonstrates how the various components interact and contribute to the efficient and secure authentication process.

#### 5.2.2. Analysis GF2

Similar to SF6-UA, GF2 is also the integration of the SF6-UA and SF9-FS, with improvements made to the labels. In GF2, the tag serves as a crucial flag. It plays a pivotal role in determining whether the one-time hash value hk requires an update. This ensures that the hash value remains fresh and secure, preventing potential security breaches due to outdated values. On the *S*’s side, the two dynamic pseudonym IDs are employed to ascertain whether the pseudonym ID and the associated value hk were updated during the previous session. By doing so, *S* can maintain an accurate and up-to-date record of the *U*’s identity and authentication status, enhancing the overall security and reliability of the communication. For a detailed security analysis of GF2, readers are referred to the literature [56].

GF2 is a two-round variant of the GF1 framework, optimized for reduced communication overhead while maintaining equivalent security properties, such as user anonymity, forward secrecy, and resistance to desynchronization. To ensure a fair comparison, we adopt the same parameter sizes used in the GF1 analysis: PID is 32 bits, ID is 32 bits, data payload (${\mathrm{Data}}_{1}$, ${\mathrm{Data}}_{2}$) is 128 bits each, and MAC is 256 bits.

In GF2, the authentication process consists of two message rounds:

**Round 1 (U → S):** $\left\{PID_{i},E_{hk}\left(ID_{i}\;∥\;{\mathrm{Data}}_{1}\right),MAC\right\}$. This requires (32+160+256)=448 bits, where $E_{hk}\left(ID_{i}\;∥\;{\mathrm{Data}}_{1}\right)$ denotes the encryption of a 32-bit $ID_{i}$ and a 128-bit ${\mathrm{Data}}_{1}$, resulting in 160 bits of ciphertext, and MAC is 256 bits.

**Round 2 (S → U):** $\left\{E_{hk}\left(PID_{inew}\;∥\;{\mathrm{Data}}_{2}\right),MAC^{\prime }\right\}$. This requires (160+256)=416 bits, where $E_{hk}\left(PID_{inew}\;∥\;{\mathrm{Data}}_{2}\right)$ consists of a 32-bit $PID_{inew}$ and a 128-bit ${\mathrm{Data}}_{2}$.

Adding these two values, the total communication cost of GF2 is 864 bits.

Compared to GF1’s total cost of 896 bits, GF2 reduces the communication overhead by 32 bits per session. This reduction is achieved by eliminating the third acknowledgment round, resulting in a lighter communication footprint while preserving the core security guarantees of the framework.

Formal proofs of GF1 and GF2’s security properties are provided in supplementary materials available at [70].

## 6. A Comparative Assessment of Existing Frameworks

In this section, we first discuss the security of the above sub-frames and general frameworks. Then, we compare the security features and performance of a series of prior related schemes.

### 6.1. Security Analysis of Existing Frameworks

**Mutual authentication:** In lightweight authentication schemes, the secret key only shared by the sender and the receiver is generally used to ensure mutual authentication. Anyone else cannot obtain this shared key. Therefore, if an authentication scheme based on any of the above frameworks can satisfy this condition, mutual authentication can be achieved.

**User anonymity:** User anonymity contains identity protection and untraceability. Identity protection means that the real identities of users can not be known by any attacker. To protect the user’s real identity, SF1-UA encrypts the real identity into ciphertext. SF2-UA, SF3-UA, SF4-UA, SF5-UA, SF6-UA, GF1, and GF2 employ pseudonym ID as a transmitted message instead of the user’s real identity. The real identity of the user is embodied in Data1 and encrypted by the secret key. Thus, it is infeasible for an adversary to revive the user’s real identity from transmitted messages without the secret key. Consequently, if the secret key is shared by all members in the system, like the second category of SF1-UA, identity protection cannot be guaranteed. Therefore, all frameworks except the second category of SF1-UA are able to support identity protection.

Untraceability means that the adversary cannot determine whether multiple messages are transmitted by the same user. SF1-UA can achieve this function by adding the current timestamp or fresh random number into ciphertext. SF3-UA, SF4-UA, SF5-UA, SF6-UA, GF1, and GF2 employ dynamic pseudonym ID to avoid being tracked. The pseudonym ID is randomly generated and changes after completing each session. Hence, it is different at each session. Therefore, all frameworks except SF2-UA can achieve untraceability.

**Forward secrecy:** Obviously, SF1-UA, SF2-UA, SF3-UA, SF4-UA, SF5-UA, and SF6-UA do not involve forward secrecy. So, schemes based on these frameworks can not provide forward secrecy. In the SF7-FS, SF8-FS, SF9-FS, GF1, and GF2, we suppose the adversary has obtained the long-term keys of participants; it still cannot revive the previous session key. The reason is that after each successful session, the keys on both sides are updated by the one-way hash function, like hk=h(hk). Due to the unidirectionality of the hash function, the adversary cannot obtain previous secret keys from the current hk. Therefore, SF7-FS, SF8-FS, SF9-FS, GF1, and GF2 can provide forward secrecy.

**Efficient GWN search operation:** Through the above analysis, the first category of SF1-UA conceals real identity into ciphertext, which makes the receiver difficult to distinguish who is the exact user. As a result, the receiver needs to search for every possible parameter or have a back-end channel to figure out the exact user. The rest of the frameworks can avoid this problem using static pseudonym ID or dynamic pseudonym ID. Therefore, all frameworks except the first category of SF1-UA are able to avoid GWN search operation.

**Resistance to desynchronization attacks:** Since SF1-UA, SF2-UA do not make use of the dynamic pseudonym ID and do not have update operations, they do not involve the problem of desynchronization. Meanwhile, although SF4-UA needs to update the secret key, it updates on only one side, and the storage of this secret key on the other side is not needed. Hence, SF4-UA does not have the problem of desynchronization. For the rest of the frameworks, SF5-UA, SF6-UA, SF8-FS, and SF9-FS are designed to resolve the problem of desynchronization in SF3-UA and SF7-FS. GF1 and GF2 are designed to achieve user anonymity, forward secrecy, and resistance to desynchronization attack at the same time. Therefore, SF5-UA, SF6-UA, SF8-FS, SF9-FS, GF1, and GF2 are able to stand against desynchronization attacks.

### 6.2. Security and Performance Comparisons

The security features comparison of nine sub-frames and two general frameworks are described in this section. As shown in Table 2, GF1 and GF2 are the only two that can fulfill the desirable security features. Therefore, GF1 and GF2 are more secure than others.

To better understand the case, we perform a security and performance assessment of 45 lightweight authentication schemes for IoT environment in Table 3. The selected schemes only use lightweight cryptographic primitives, like symmetric key encryption/decryption or hash functions. From Table 3, it is easy to see that only four schemes meet all the security requirements, including Nashwan et al.’s scheme [57], Xiong et al.’s scheme [55], Xiong et al.’s scheme [56], and Gope et al.’s scheme [50]. Gope et al.’s scheme [50] achieves forward secrecy using a one-way function named physically unclonable function (PUF), which consists of integrated circuits (ICs) that cannot be duplicated. This work does not make an in-depth analysis of PUF. The other two schemes proposed by Xiong et al.’s [55,56] based on GF1 and GF2 meet all security requirements. Meanwhile, the performance of these three schemes differs slightly. Therefore, lightweight authentication schemes based on G1 and G2 have better security than others.

## 7. Conclusions

This paper presented a systematic review and taxonomy of lightweight anonymous authentication for the Internet of Things. We deconstructed the landscape of existing protocols into nine fundamental sub-frameworks, categorizing them by their approach to achieving user anonymity and forward secrecy. Building on this classification, we synthesized two general frameworks, GF1 and GF2, that concurrently provide both security properties while addressing the critical challenge of desynchronization attacks. A comparative analysis of our frameworks alongside 45 representative schemes highlighted the inherent trade-offs between security guarantees, communication overhead, and resilience. Ultimately, this work provides a structured understanding of the field and offers robust, reusable blueprints to guide the design of more secure and efficient IoT authentication protocols.

Building on this analysis, several critical directions for future research emerge. First, there is a pressing need for empirical validation; future work should focus on implementing promising theoretical frameworks on real-world hardware (e.g., FPGAs, ASICs) to benchmark their practical latency, power, and resource costs. Second, ensuring long-term security against quantum threats is paramount; integrating lightweight post-quantum cryptography (PQC) into existing IoT authentication models is a vital research avenue. Third, moving beyond centralized trust models by exploring decentralized architectures using blockchain and DIDs could resolve many existing bottlenecks. Finally, as PUF-based solutions become more prevalent, a deeper investigation into their resilience against advanced machine learning-based modeling attacks and the development of corresponding countermeasures is essential for ensuring their continued viability.

## Figures and Tables

**Table 1:** Notations.

Notation	Descriptions
*U*	The sender refers to $U_{i}$ or GWN in Figure 3
*S*	The receiver refers to GWN or $S_{j}$ in Figure 3
$ID_{i}$	The real identity of *U*
$PID_{i}$	The pseudonym identity of *U*
Ki	The secret key to protect $ID_{i}$, which is the shared key between *U* and *S* or group shared key
$K_{us}$	The secret key shared by *U* and *S*
Data1	The messages which *U* intended to send to *S*
MAC	The messages authentication value, which is generated accord to Section 3.5
Data2	The messages which *U* intended to transmit to *S*
$MAC^{\prime }$	The returned message authentication value, which is generated according to Section 3.5
$PID_{inew}$, $PID_{iold}$	Two pseudonym IDs of *S*
*h*	The general one-way hash function
hk	The one-time secret key shared by *U* and *S*, which is updated by hash function
Nui,Nsj	The serial number of *U* and *S*, respectively, which represents the number of times of performing one-time hash chain value
$f_{0}$,$f_{1}$	The tag of *U* and *S*, respectively
||	String concatenation operation
⊕	XOR operation

**Table 2:** Security features comparisons of existing frameworks.

	SF1		SF2	SF3	SF4	SF5	SF6	SF7	SF8	SF9	GF1	GF2
	1	2										
R1	√	√	√	√	√	√	√	√	√	√	√	√
R2	√	×	×	√	√	√	√	−	−	−	√	√
R3	−	−	−	−	−	−	−	√	√	√	√	√
R4	×	√	√	√	√	√	√	√	√	√	√	√
R5	−	−	−	×	√	√	√	×	√	√	√	√

R1, R2, R3, R4, R5 represent mutual authentication, user anonymity, forward secrecy, efficient GWN search operation, and resistance to desynchronization attack, respectively. SF1∼SF9 denote SF1-UA, SF2-UA, SF3-UA, SF4-UA, SF5-UA, SF6-UA, SF7-FS, SF8-FS, SF9-FS, respectively; 1 and 2 denote two categories of SF1-UA. √/× denotes the framework can/cannot provide the corresponding security requirement. − denotes the framework does not involved the corresponding security requirement.

**Table 3:** Security features comparisons of our scheme and other relate schemes.

Scheme	Year	Framework	Security Features					Performance		
			R1	R2	R3	R4	R5	User	GWN	Sensor
Liu [71]	2025	SF4-UA	√	√	√	√	×	$8T_{h}$	$11T_{h}$	−
Xie [72]	2024	SF4-UA	√	√	−	√	√	$7T_{h}$	$6T_{h}$	$2T_{h}$
Luo [38]	2020	SF3-UA + SF7-FS	√	√	√	√	×	$8T_{h}$	$11T_{h}$	$5T_{h}$
Wazid [39]	2020	SF3-UA	√	√	−	√	×	$9T_{h}+T_{E}$	$10T_{h}+2T_{E}$	$6T_{h}+T_{E}$
Nashwan [57]	2020	GF1	√	√	√	√	√	$4T_{h}+2T_{E}$	$10T_{h}+2T_{E}$	$4T_{h}$
Shuai [40]	2020	SF3-UA + SF8-FS	√	√	√	√	×	$9T_{h}$	$12T_{h}$	$6T_{h}$
Yang [25]	2020	SF2-UA + SF9-FS	−	√	√	√	√	$10T_{h}$	$19T_{h}$	$8T_{h}$
Banerjee [41]	2020	SF3-UA	√	√	−	√	×	$10T_{h}$	$10T_{h}$	$4T_{h}$
Fakroon [19]	2020	SF1-UA	√	√	−	×	√	$4T_{h}$	$4T_{h}$	$3T_{h}$
Banerjee [67]	2019	SF4-UA	√	√	−	√	√	$12T_{h}+3T_{E}$	$5T_{h}+5T_{E}$	$2T_{h}+2T_{E}$
Xiong [56]	2019	GF2	√	√	√	√	√	$8T_{h}$	$10T_{h}$	$4T_{h}$
Shuai [58]	2019	SF1-UA + SF8-FS	√	√	√	×	√	$11T_{h}$	$12T_{h}$	$7T_{h}$
Gope [50]	2019	SF5-UA	√	√	√	√	√	$6T_{h}+3T_{P}$	$9T_{h}$	$4T_{h}+2T_{P}$
Ostadsharif [30]	2019	SF2-UA	√	×	−	×	√	$11T_{h}$	$17T_{h}$	$5T_{h}$
Wazid [21]	2018	SF1-UA	√	√	−	×	√	$13T_{h}+2T_{E}$	$5T_{h}+4T_{E}$	$4T_{h}+2T_{E}$
Ali [42]	2018	SF3-UA	√	√	−	√	×	$11T_{h}+2T_{E}$	$16T_{h}+3T_{E}$	$6T_{h}+T_{E}$
Chen [22]	2018	SF1-UA	√	×	−	√	√	$11T_{h}$	$11T_{h}$	$4T_{h}$
Amin [43]	2018	SF3-UA	√	√	−	√	×	$12T_{h}$	$16T_{h}$	$6T_{h}$
Amin [47]	2017	SF4-UA	√	√	−	√	√	$14T_{h}$	$17T_{h}$	$4T_{h}$
Xiong [55]	2017	GF1	√	√	√	√	√	$9T_{h}+2T_{E}$	$11T_{h}+2T_{E}$	$4T_{h}$
Mohit [18]	2017	SF1-UA	√	×	−	√	√	$7T_{h}$	$9T_{h}$	$4T_{h}$
Wu [48]	2017	SF4-UA	√	√	−	√	√	$11T_{h}$	$17T_{h}$	$6T_{h}$
Dhillon [31]	2017	SF2-UA	√	×	−	√	√	$8T_{h}$	$6T_{h}$	$8T_{h}$
Tai [15]	2017	SF1-UA	√	×	−	√	√	$8T_{h}$	$10T_{h}$	$6T_{h}$
Srinivas [26]	2017	SF2-UA	√	×	−	√	√	$10T_{h}$	$13T_{h}$	$6T_{h}$
Li [32]	2017	SF2-UA	√	×	−	√	√	$10T_{h}$	$8T_{h}$	$2T_{h}$
Wu [49]	2017	SF4-UA	√	√	−	√	√	$9T_{h}$	$11T_{h}$	$4T_{h}$
Wu [68]	2017	SF4-UA	√	√	−	√	√	$10T_{h}+2T_{E}$	$6T_{h}+5T_{E}$	$4T_{h}+T_{E}$
Lu [23]	2016	SF1-UA	√	√	−	×	√	$7T_{h}+2T_{E}$	$8T_{h}+4T_{E}$	$4T_{h}+2T_{E}$
Jung [24]	2016	SF1-UA	√	√	−	×	√	$5T_{h}+2T_{E}$	$5T_{h}+2T_{E}$	$4T_{h}$
Gope [11]	2016	SF5-UA + SF7-FS	√	√	√	√	×	$11T_{h}$	$9T_{h}$	$4T_{h}$
Das [45]	2016	SF3-UA	√	√	−	√	×	$9T_{h}$	$11T_{h}$	$5T_{h}$
Amin [27]	2016	SF2-UA	√	×	−	√	√	$7T_{h}$	$8T_{h}$	$5T_{h}$
Chang [28]	2016	SF2-UA	√	×	−	√	√	$7T_{h}$	$8T_{h}$	$5T_{h}$
Kumari [29]	2016	SF2-UA	√	×	−	√	√	$10T_{h}$	$8T_{h}$	$6T_{h}$
Vaidya [16]	2016	SF1-UA	−	×	−	√	√	$8T_{h}$	$6T_{h}$	$3T_{h}$
Jiang [44]	2015	SF3-UA	√	√	−	×	×	$7T_{h}$	$10T_{h}$	$5T_{h}$
Chang [54]	2015	SF6-UA	√	√	−	√	×	$11T_{h}$	$10T_{h}$	$4T_{h}$
He [34]	2015	SF2-UA	√	×	−	√	√	$6T_{h}$	$10T_{h}$	$7T_{h}$
Turkanovic [35]	2014	SF2-UA	√	×	−	√	√	$7T_{h}$	$7T_{h}$	$5T_{h}$
Kim [36]	2014	SF2-UA	√	×	−	√	√	$8T_{h}$	$8T_{h}$	$2T_{h}$
Xue [37]	2013	SF2-UA	√	×	−	√	√	$7T_{h}$	$10T_{h}$	$5T_{h}$
Kumar [17]	2012	SF1-UA	−	×	−	√	√	$4T_{h}+2T_{E}$	$T_{h}+3T_{E}$	$T_{h}+2T_{E}$
Kumar [33]	2011	SF2-UA	√	×	−	√	√	$4T_{h}+2T_{E}$	$5T_{h}+2T_{E}$	$2T_{h}+2T_{E}$
Das [14]	2009	SF1-UA	−	×	−	√	√	$4T_{h}$	$4T_{h}$	$T_{h}$

R1, R2, R3, R4, R5 represent mutual authentication, user anonymity, forward secrecy, effective GWN search operation, and resistance to desynchronization attack, respectively. *T*_*h*_, *T*_*E*_, *T*_*P*_ denote the time complexity of the general hash operation, symmetric encryption/decryption operation, and physically unclonable function (PUF) operation. Some lightweight operations like XOR and connection operation are omitted. √/× denotes the schemecan/cannot provide the corresponding security requirement. − denotes the framework does not involve the corresponding security requirement.

## Data Availability

No new data were created or analyzed in this study.
